# Global Disparities in Hepatitis B Elimination—A Focus on Africa

**DOI:** 10.3390/v14010082

**Published:** 2022-01-03

**Authors:** Mark W. Sonderup, C. Wendy Spearman

**Affiliations:** Division of Hepatology, Department of Medicine, Faculty of Health Sciences, University of Cape Town and Groote Schuur Hospital, Cape Town 7925, South Africa; wendy.spearman@uct.ac.za

**Keywords:** disparities, viral hepatitis, elimination, Hep B birth dose, vaccination, integration, triple elimination

## Abstract

In 2016, WHO member states at the World Health Assembly adopted a Global Health Sector Strategy that included a policy of eliminating viral hepatitis. Clear targets were established to assist in achieving this by 2030. The strategy, while achievable, has exposed existing global disparities in healthcare systems and their ability to implement such policies. Compounding this, the regions with most disparity are also those where the hepatitis B prevalence and disease burden are the greatest. Foundational to hepatitis B elimination is the identification of both those with chronic infection and crucially pregnant women, and primary prevention through vaccination. Vaccination, including the birth dose and full three-dose coverage, is key, but complete mother-to-child transmission prevention includes reducing the maternal hepatitis B viral load in the third trimester where appropriate. Innovations and simplified tools exist in order to achieve elimination, but what is desperately required is the will to implement these strategies through the support of appropriate investment and funding. Without this, disparities will continue.

## 1. Introduction

Globally, an estimated 296 million people are chronically infected with hepatitis B virus (HBV) [1]. In 2019, approximately 1.5 million new HBV infections occurred, with a corresponding 820,000 [95% CI 450,000–950,000] deaths attributable to HBV-related cirrhosis or hepatocellular carcinoma [1]. Overall, an estimated 10% of those chronically infected have been diagnosed. Importantly, the vast majority of those diagnosed and on treatment live in the Americas, Europe, Asia, or the Western Pacific regions. In Africa, less than 2% are diagnosed and a paltry 0.1% are on treatment. This contrasts sharply with HBV prevalence in Africa, where it is endemic, with a 60% lifetime risk of acquiring HBV infection. An estimated 82 million people are chronically infected, representing one-quarter of the globally infected population. Table 1 denotes the estimated HBsAg seroprevalence of sub-Saharan African countries based on the WHO viral hepatitis scorecard data [2]. A total of 30 of the 47 WHO AFRO region countries have a HBsAg prevalence >5% and 70% of all new HBV infections occur in the WHO AFRO region. Moreover, more than two-thirds of the now 6.3 million children below the age of 5 who are hepatitis-B-surface-antigen-positive (HBsAg) reside in the WHO AFRO region [3].

In 2016, the World Health Organization set the policy goal through a global health sector strategy of eliminating viral hepatitis, including both hepatitis B and C, by 2030 [4]. The effect would be a 90% global reduction in new infections and a consequent 65% reduction in mortality. Several targets were set for hepatitis B that aimed to achieve elimination, with the elimination set at a HBsAg prevalence in children under five years old of <1.0% by 2020 and ≤0.1% by 2030 [5]. The WHO AFRO region goal for 2020 was <2%. To achieve this, several policies are required to be implemented by individual countries. Given the variance in global hepatitis B prevalence, many countries will be able to achieve these targets for HBV and several are already on target. It is now evident that, of the 32 countries projected to have an HBsAg prevalence of >1% in 2030, 26 are in sub-Saharan Africa [6,7]. Globally, the prevention of mother-to-child transmission programs, timely birth dose vaccination, full HBV vaccination coverage, diagnosis, and linkage to care and treatment is varied, but is particularly worrying in world regions with the greatest prevalence of chronic HBV (central, east, and south-east Asia, sub-Saharan Africa), where the lowest rates of HBV diagnosis and treatment exist. If progress is to be achieved, a vast amount of multilateral effort, funding, and, importantly, government investment and will is required.

## 2. Disparities in Eliminating Mother-to-Child and Early Childhood Transmission

In hyperendemic regions of the world, HBV chronicity is established in early childhood in the absence of an effective prevention of mother-to-child-transmission (PMTCT), timely hepatitis B birth dose vaccination, and a full three-dose HBV vaccine coverage. This is confirmed by data demonstrating no difference in HBsAg seroprevalence between children aged 5–9 years and adults in these hyperendemic regions [8]. The risk of developing chronic HBV infection is inversely related to the age of acquisition of infection, with 90% risk after neonatal infection, 20–50% risk with childhood infection (<5 years of age), and <5% for adults aged >20 years. In sub-Saharan Africa, horizontal transmission in children between the ages of 6 months and 5 years is typical, given close interactions between infected household contacts and playmates. A phylogenetic analysis in The Gambia confirmed intra-familial HBV transmission in at least two-thirds of families studied [9]. Vertical MTCT transmission from highly viremic (HBV DNA >200,000 IU/mL, typically HBeAg positive) pregnant women can also occur in sub-Saharan Africa, although this is the more typical transmission route in Asia. Importantly, HIV-HBV co-infection significantly increases the risk of MTCT if antiviral therapy is not administered to infected mothers [10]. Incident chronic infection in neonates implies a failure of maternal and child healthcare programs to prevent mother-to-child (MTCT) or early childhood transmission [11]. Perinatal and childhood HBV acquisition not only has an enhanced risk of chronicity, but also strongly predicts worse long-term outcomes for liver cirrhosis and hepatocellular carcinoma [12]. Longitudinal data from The Gambia also confirmed the markedly elevated risks of chronic liver disease and associated complications in those perinatally infected [13]. This underpins the crucial importance of interrupting perinatal transmission.

Hepatitis B is a wholly vaccine-preventable chronic viral infection with an effective vaccine available for more than 4 decades [14]. To date, 196 countries globally and, specifically, 47 countries in the WHO AFRO region, have introduced HBV vaccinations into their routine infant immunization schedules, with 44 (94%) countries using the pentavalent vaccine (diphtheria, tetanus, pertussis, Haemophilus influenza type B, and hepatitis B vaccines) and 33 (70%) countries following a three-dose schedule at 6, 10, and 14 weeks of age [15]. The estimated median full three-dose coverage (HepB3) in the African region is a concerning 77%—compared to a range of 81% (Eastern Mediterranean region) to 95% in the Western Pacific region, with a median global average of 84% [16]. The net effect is that vaccination has had a substantial impact upon global HBsAg seroprevalence. Modeled data from 1980 to 2007 demonstrate a global decline in HBsAg prevalence in children <5 years, from 4·7% in the pre-vaccine implementation period to 1·3% in 2015 [11]. In 2019, it was estimated to be 0.94 [UI 0.82–1.06] [1]. However, with a poor uptake of HB-BD vaccination and an incomplete HBV three-dose vaccine coverage, HBsAg prevalence in the WHO AFRO region in 2019 remains high at 2.53% [UI 2.1–3.07], with 360 000 children infected every year [1,17].

In 2009, the WHO advised the introduction of a birth dose of the hepatitis B vaccine (HepB-BD), administered within 24 h of delivery, the effect being to mitigate the mother-to-child transmission risk. A timely birth dose effectively reduces this risk by 90% [18]. Despite the 2009 recommendation, the global scale-up of a timely HepB-BD has been slow, with variable coverage and no financial or funding support from organizations such as GAVI. An estimated 43% of newborns in 110 countries in 2019 received a timely HepB-BD vaccine. In the WHO AFRO region, 14 countries have adopted HepB-BD policies, but coverage in the region is a disappointing 7% [3]. In 2019, GAVI re-evaluated its funding support for the timely HepB-BD vaccination, but these efforts have been thwarted by the COVID-19 pandemic [19].

Recently, modeled data suggested that, in the absence of a scaled up HepB-BD plus HepB3, the elimination target of HBsAg < 0.1% in those under 5 years of age would be achieved in the WHO EURO region in 2037, followed by the Americas in 2042. Both these regions already have timely HepB-BD coverage and low HBV seroprevalence. Comparatively, the African and Eastern Mediterranean WHO regions would not achieve this before 2100 [3]. An attempt to scale up timely HepB-BD vaccination coverage to 90% by 2030 (a WHO elimination target) would have the effect of an immediate reduction in incident chronic HBV cases and HBsAg prevalence in under-5-year-olds. In terms of HBV-related mortality in the 2020 to 2030 birth cohorts, the effect globally would be a reduction of an estimated 710,000 [580,000 to 890,000] deaths. In WHO AFRO, where, currently, the HepB-BD coverage is very low, a more realistic scale up of timely HepB-BD to ≥25% by 2030 would avert 150,000 [120,000 to 190,000] deaths in this birth cohort.

Given the low coverage of HepB-BD in Africa, a goal-directed upscaling would have a greater effect and the benefit would be more significant. Notably, even at scaled-up rates, the 2030 target for most regions is not achievable and, for Africa, 2060 is more realistic. The challenges of scaling up HepB-BD are not insubstantial but are manageable. COVID-19 has clearly had a negative effect, but the need to administer doses within 24 h does pose practical challenges, especially in rural areas. Easy to use, task-shifted (birth attendants), and disposable injecting devices, such as the hepatitis B Uniject^R^, presents a possible solution [20]. The need for maintaining a cold chain is also mitigatable, as the vaccine is potentially heat-stable for a limited period [21].

The disparities in the HBV birth dose and full HepB3 vaccine coverage underpin the obvious differences in the predicted likelihood of achieving elimination targets. HBV prevalence (HBsAg positivity) in pregnant women (and year of data analysis) varies geographically in Africa, with 3.7% in Rwanda (2017), 6.0% Malawi (2016), 4.4% Cameroon (2016), 10.7% Mauritania (2012), 8% Mali (2011), 5.6% South Sudan (2017), and 9.2% in The Gambia (2019) [22]. The HBsAg prevalence in a meta-analysis of 30 African countries was 6.8% [95% confidence interval: 6.1–7.6] As a surrogate marker of a high HBV viral load, the HBeAg positive prevalence in pregnant women who were HBsAg-positive, was 18.9% [95% CI: 14.4–23.9] [22]. Vaccination programs were initially guided by a perceived lower risk of perinatal transmission from a predominant population of HBeAg-negative, anti-HBe-positive childbearing women in sub-Saharan Africa [23]. These data suggest that, even with HepB-BD plus HepB3, vaccination would not be an adequate strategy to drive HBsAg seroprevalence down to <0.1% in under-5-year-olds. While vaccination is the foundation upon which transmission prevention rests, a complete package of care to minimize the perinatal transmission risk is needed. Following seminal studies and data, WHO now recommends that all pregnant women be screened for HBsAg as part of antenatal care [24]. Pregnant women with a HBV DNA viral load of >200,000 IU/mL (≥5.3 log10 IU/mL) should receive tenofovir prophylaxis from the 28th week of pregnancy until at least birth in order to augment the risk reduction in the mother-to-child transmission of HBV. This is in addition to the timely birth dose followed by three doses of the HBV vaccination. In settings where HBV DNA testing is unavailable, HBeAg testing may be used as a surrogate marker instead of HBV DNA testing to enable eligibility for tenofovir prophylaxis [24]. The efficacy of tenofovir in this setting has recently been supported in an extensive systematic review and meta-analysis [25]. At present, only three countries in Africa—Cameroon, Rwanda, and Mauritania—have national guidelines addressing the mother-to-child transmission of hepatitis B [23]. The challenges of successfully implementing such a program are considerable. These include the availability of HBsAg, HBeAg testing, affordable access to HBV DNA quantification, and a ready supply of tenofovir for HBV mono-infected persons that is specifically available for this purpose. The disparity is accentuated by HIV-HBV co-infected women inadvertently accessing antiretroviral therapy as part of significantly upscaled HIV testing and treatment. Many would receive tenofovir-lamivudine or tenofovir-emtricitabine as part of their ART regimens, appropriately providing them with both the HIV and HBV PMTCT benefit. Women who are HBV-mono-infected are frequently unable to access tenofovir.

A potential solution to the slow pace of these programs is an integrated approach where the triple elimination of HIV, hepatitis B, and syphilis is carried out in tandem in order to attain the targets of a reduced mother-to-child transmission. Barriers and disparities remain and include a lack of political will, poor availability, and access to affordable and preferably point-of-care diagnostics and sustainable drug supplies of benzathine penicillin and tenofovir [26]. The three infections do have some commonalities in their acquisition and transmission risk. They are all associated with a considerable disease burden in Africa and, to an extent, their presence is synergistic in enhancing the transmission risk. Their diagnosis is relatively simple, and effective interventions exist to reduce or negate transmission [26]. To achieve integration, the WHO recommendation to screen all pregnant women for HIV, syphilis, and hepatitis B is of primary importance [24]. In addition, clear normative guidance exists for the perinatal management of these infections. Combined rapid diagnostic tests for HIV, syphilis, and the hepatitis B virus are an aid to integrate screening in antenatal settings, with several products available, although none are WHO-prequalified yet. Countries such as China have already used their HIV PMTCT program to enhance the antenatal prevention of syphilis and hepatitis B mother-to-child transmission and have demonstrated its efficiencies [27]. This was achieved through layering in a combined infection screening to an existing service without destabilizing the service, and instead capacitating and resourcing it appropriately. This approach may well be the solution to overcome the disparities in Africa, where funding has supported and ensured a significant upscaling of HIV PMTCT programs, in addition to HIV care in general. What would be required is the will to integrate HBV and syphilis testing and the subsequent linkage to appropriate care. None of these interventions are prohibitively costly but do require dedicated investment. Data do support these interventions as being highly cost-effective, with an ICER ratio of USD 114 per DALY averted [28].

## 3. Disparities in the Treatment of Chronic Hepatitis B Virus Infection

Most HBV treatment guidance is based on the presence and degree of fibrosis (or cirrhosis) or a combination of abnormal serum transaminases (upper limits vary), HBV DNA quantification ranging from 2000 IU/mL to 20,000 IU/mL, histological parameters, and the assessment of liver fibrosis by non-invasive methods, including transient elastography (e.g., Fibroscan^R^), the AST-to-platelet ratio index (APRI) or the fibrosis-4 score (FIB-4). The aim is to identify patients who would benefit from oral nucleoside therapy, such as tenofovir or entecavir, which aims to suppress HBV replication and achieve long-term morbidity, and even mortality benefits. It is important to note that no treatment guidance has been validated for African patients and, theoretically at least, the same thresholds may not be applicable [29]. Furthermore, no data exist for Africans and whether long-term HBV DNA suppression ameliorates HCC risk. Even so, it would be irrational not to treat African patients who meet present guidance criteria.

Most guidance incorporates HBV DNA quantification, which, at present, is unaffordable and unavailable to many in Africa. WHO guidance advised a pragmatic treatment algorithm where HBV DNA quantification is unavailable, which instead utilizes persistently elevated aminotransferase levels, the APRI score, and evidence of cirrhosis by clinical stigmata [30]. HBeAg, as a surrogate marker of high HBV DNA levels, is also an alternate to HBV DNA measurement. Prospective data from Ethiopia highlighted the relative lack of sensitivity of the WHO guidance when benchmarked against EASL or AASLD HBV treatment guidelines [31]. Biomarker-based tests, such as the APRI score, also underestimate cirrhosis in Africa, with their performance potentially not adequate enough [32]. Transient elastography provides a real-time fibrosis assessment and its performance is better than those of APRI or FIB-4 in an African setting [33]. However, the technology remains costly and its availability across the African region is limited. To contextualize this disparity, there are 52 Fibroscan^R^ machines available in sub-Saharan Africa and several hundred in North Africa. In contrast, the United Kingdom and France individually have more than 400 machines each (personal communication, Echosens, Paris, France). It is these disparities that require innovative solutions to ensure that patients with chronic HBV can access this level of care, but, equally, for governments, policy makers, and funders to understand the need for investment.

There are several solutions to the disparity in the availability of HBV DNA quantification. As noted, the upscaling of the efforts against HIV in the global epicenter of the HIV pandemic, sub-Saharan Africa, has created infrastructures such as the widespread availability of platforms to perform the HIV RNA assessment. This has been used to great effect in Rwanda, where the available GeneXpert^R^ platforms (Cepheid, Sunnyvale, CA, USA) were capacitated to include a hepatitis B and C virological measurement for their progressively upscaled viral hepatitis treatment program [34]. Such infrastructure exists elsewhere in sub-Saharan Africa—the difference in Rwanda was the political will, strategic partnerships, and the progressive execution of a national plan. Alternatives to HBV DNA quantification may be another solution. In this respect, the hepatitis B core-related antigen (HBcrAg) is a potentially more affordable alternative. Data from a patient cohort in The Gambia suggest that the AUROC, sensitivity, and specificity of serum HBcrAg was 0.88 (95% CI; 0.82–0.93), 83.3%, and 83.9%, respectively, to diagnose HBV DNA ≥2000 IU/mL and 0.94 (95% CI; 0.88–0.99), 91.4%, and 93.2% for ≥200,000 IU/mL [35]. Consequently, using a simplified treatment approach, HBcrAg alone demonstrated an AUROC of 0.91 [95% CI; 0.88–0.95]) with a sensitivity of 96.6% and specificity of 85.8%. HBcrAg still requires laboratory infrastructure, but it may be more affordable, and evidence exists that validates its performance using dried blood spot testing [36]. There is now supportive evidence for its value in discriminating those in the immune control/chronic infection versus immune reactive/chronic hepatitis phase of infection in HBeAg-negative patients [37]. HBcrAg may thus offer a useful and possibly more cost-effective alternative in deciding who warrants therapy, thus avoiding unneeded treatment in a test and treat approach, as has been suggested by some [38].

Preceding treatment, identifying those with chronic HBV is the fundamental inflection point for those infected to be identified and then linked to assessment. Detecting cases is thus pivotal. Most patients are unaware of their diagnosis, with fewer than 10% diagnosed and <2% on treatment. Although, formal laboratory testing for HBsAg is available, it is not practical to achieve the level of upscaling required. WHO pre-qualified HBV rapid diagnostic tests for HBsAg include Determine HBsAg 2, Vikia HBsAg, and SD BIOLINE HBsAg WB. Furthermore, a number of point-of-care HBsAg rapid diagnostic tests have been validated in Africa. These include the Alere Determine HBsAg [Alere, Waltham, MA, USA], VIKIA HBsAg [BioMérieux, Craponne, France], and Espline HBsAg [Fujirebio Inc., Tokyo, Japan]. They were assessed in the PROLIFICA study in The Gambia [39]. The sensitivity and specificity of the Determine test was 88·5% and 100% in the field and 95·3% and 93·3% in a laboratory setting. The Vikia test had a 90% sensitivity and 99·8% specificity in the field, and Espline had a 93·9% sensitivity and 94·7% specificity in the laboratory [40].

Opportunities exist to overcome the disparities of screening—what is required is to prioritize and implement upscaled screening and treatment. The disparate issue here is simply the lack of impetus to implement such screening.

In terms of drug therapy, the base price of currently available generic nucleoside antivirals, such as tenofovir, is no longer a limiting factor for the elimination of hepatitis B in Africa. However, it is not always accessible or available to mono-infected HBV patients given its use in externally funded HIV programs. Tenofovir alafenamide, a prodrug of tenofovir disiproxil fumarate (TDF), has been approved for the treatment of chronic hepatitis B in adults with compensated liver disease in many countries outside of Africa. It is non-inferior to TDF but has a better side effect profile with respect to long-term bone mineral density and creatinine clearance [41]. Despite the cost of tenofovir having declined, dedicated funding for tenofovir for HBV-mono-infected patients remains elusive and maintains the disparity in care for those with chronic HBV infection.

## 4. Conclusions

Disparities in the elimination of hepatitis B globally are rife. Many countries are already at or have achieved the important target of an HBsAg seroprevalence of <0.1% in under-5-year-olds. The paradox is that regions of a very high HBV burden, such as sub-Saharan Africa, are not remotely on track to achieve this target in the medium- or even long-term at their current pace. The irony is that many opportunities, policy options, and tools exist to enhance and upscale hepatitis B care. While proper funding and investment remains elusive, a fundamental problem is the lack of will and intransigence to implement. Regional policy decisions, such as the Cairo Declaration from the African Union in 2019, allow for a high level of political support [42]. COVID-19 has been a significant setback in advancing this, but, simultaneously, has focused a spotlight on what can be achieved if need and will are present. Champion countries, such as Rwanda, have demonstrated what can be achieved. It is apparent that community involvement is essential to mobilize resources, create demand, improve knowledge, and access treatment, which is something that the HIV experience has taught us. Until such a time, disparities will persist.

## Figures and Tables

**Table 1:** HBsAg sero-prevalence (https://www.afro.who.int/publications/hepatitis-scorecard-who-africa-region-implementing-hepatitis-elimination-strategy (accessed on 21 December 2021)).

Country	HBsAg Sero-Prevalence
Algeria	1.8
Angola	9.4
Benin	11
Botswana	1.3
Burkina Faso	10.1
Burundi	6.4
Cabo Verde	5.2
Cameroon	4.4
Central African Republic	10.2
Chad	4.9
Comoros	4.3
Congo	9.5
Cote-d’Ivore	6.1
Eswatini	3.2
Equatorial Guinea	9.3
Eritrea	1.9
Ethiopia	5.7
Gabon	9.1
The Gambia	5.8
Ghana	8.6
Guinea	13
Guinea Bissau	5.1
Kenya	2.2
Lesotho	4.5
Liberia	14.9
Madagascar	8.2
Malawi	6.9
Mali	8.5
Mauritania	10.2
Mauritius	1.9
Mozambique	7.2
Namibia	2.2
Niger	11.6
Nigeria	5.5
Rwanda	4.5
São Tomé and Principe	5.5
Senegal	8.2
Seychelles	0.4
Sierra Leone	8.6
South Africa	6.1
South Sudan	22.2
Tanzania	4
Togo	8.4
Uganda	6.3
Zambia	4.1
Zimbabwe	10.1
